# Associations between sex steroid hormones and female malignancies: A cross-sectional analysis of NHANES data

**DOI:** 10.1097/MD.0000000000050317

**Published:** 2026-08-21

**Authors:** Ningning Ren, Mengjie Yang, Fangyu Zhou, Jiamin Ma, Wenzhong Ji, Xiaoyi Ren

**Affiliations:** a Department of Breast Surgery, The Second Affiliated Hospital of Zhengzhou University, Zhengzhou, Henan, China.

**Keywords:** female malignancies, machine learning models, NHANES, sex steroid hormones

## Abstract

Sex steroid hormones, including estradiol (E2), estrone (E1), follicle-stimulating hormone (FSH), and luteinizing hormone (LH), have been implicated in the development of female malignancies (FMs), yet their independent and combined effects remain unclear. We conducted a cross-sectional analysis using data from the National Health and Nutrition Examination Survey (NHANES). Weighted logistic regression, restricted cubic spline modeling, and chi-square trend tests were applied to evaluate dose-response associations. Machine learning algorithms, including Bayesian Kernel Machine Regression, Extreme Gradient Boosting, Shapley Additive Explanations, Least Absolute Shrinkage and Selection Operator regression, and Random Forest, were used to explore nonlinear relationships and assess variable importance in an exploratory manner. Sensitivity analyses confirmed the robustness of the findings. A total of 2108 participants were included. Higher FSH and LH levels were associated with increased odds of FMs, whereas higher E2 levels were associated with lower odds. In exploratory machine learning analyses, FSH and E1 were consistently ranked as important predictors of FMs. In this cross-sectional analysis, LH and FSH were associated with increased odds of FM, while higher E2 levels were inversely associated with malignancy risk. Exploratory machine learning analyses highlighted additional nonlinear patterns involving E2 and E1.

## 1. Introduction

Female malignancies (FMs) have emerged as a major global public health concern, posing a significant threat to women’s health. According to the Global Cancer Burden Report 2020 released by the International Agency for Research on Cancer, there were over 9 million new cancer cases among women worldwide.^[1]^ Cervical cancer, ovarian cancer, and endometrial cancer exhibit high incidence and mortality rates among women. The development of these cancers is influenced by a complex interplay of factors, including genetic susceptibility, lifestyle behaviors, environmental exposures, metabolic status, and endocrine regulation.^[2–5]^ Notably, in perimenopausal and postmenopausal women, fluctuations in endogenous hormone levels are associated with an increased risk of developing certain hormone-related malignancies.^[6]^

**Figure 1.**
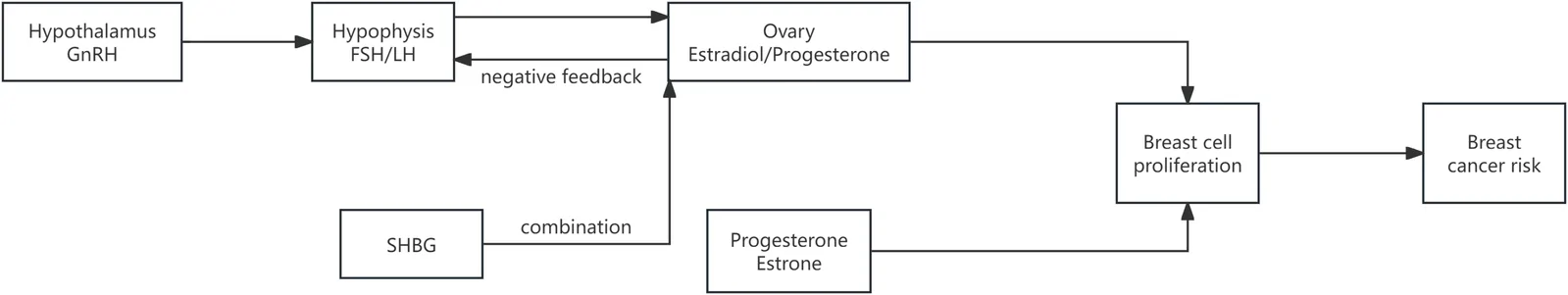
Mechanistic role of sex steroid hormones in the development of breast cancer. FSH = follicle-stimulating hormone, LH = luteinizing hormone.

In women, sex steroid hormones (SSHs) primarily include estrogens, such as estradiol (E2) and estrone (E1), as well as gonadotropins, including follicle-stimulating hormone (FSH) and luteinizing hormone (LH).^[7]^ E1 is the predominant form of circulating estrogen in postmenopausal women; it exhibits relatively weaker estrogenic activity compared to E2.^[8]^ LH and FSH indirectly regulate the synthesis and secretion of ovarian estrogens.^[9,10]^ Evidence suggests that prolonged exposure to elevated estrogen levels may increase the risk of tumorigenesis (Fig. 1).^[11]^

Moreover, these 4 hormones do not act independently within the body but rather participate in a dynamic feedback regulatory network.^[12]^ This regulatory network gives rise to significant correlations and potential interactions among the 4 hormones. Existing literature has largely focused on the impact of individual hormones on cancer risk, with limited exploration of hormone–hormone interaction patterns. Therefore, evaluating these hormones jointly may better reflect real-world endocrine milieus than single-hormone analyses, particularly in population-based studies. Given the above background, systematically evaluating the relationship between endogenous hormone levels and FMs, particularly their potential nonlinear effects and interactive mechanisms, holds significant scientific and clinical relevance.

This study aimed to investigate the associations between E2, E1, FSH, and LH levels and the occurrence of FMs using data from the National Health and Nutrition Examination Survey (NHANES). NHANES employs standardized chemical analytical methods to quantify hormone concentrations. Data from different survey cycles were weighted and adjusted to ensure comparability, thereby enabling population-level epidemiological analyses of hormone exposures. Compared with previous studies, this study simultaneously evaluated the comprehensive effects of E2, E1, FSH, and LH in nationally representative samples by integrating traditional statistical methods with machine learning approaches to explore nonlinear patterns and relative variable importance, while emphasizing association-based interpretation.^[7]^

## 2. Methods

### 2.1. Data sources and study population

The NHANES is a large-scale, cross-sectional survey conducted across the United States and administered by the National Center for Health Statistics, a division of the Centers for Disease Control and Prevention. Its primary goal is to systematically evaluate the health and nutritional status of the U.S. population using standardized protocols and data collection procedures. This study adopted a cross-sectional design based on NHANES’s multistage, stratified sampling strategy. Weighted analytical methods were employed to produce statistically valid, nationally representative estimates reflective of the broader U.S. population. This study was conducted in accordance with the STROBE guidelines.

Data were extracted from 2 consecutive cycles of the NHANES dataset: 2017 to 2020 and 2021 to 2023 (https://wwwn.cdc.gov/nchs/nhanes/). All variables included in the analysis were consistently available in both survey cycles. Data from different survey cycles were weighted and standardized to ensure the comparability and consistency of variables. The primary inclusion criteria were as follows: female participants; adults aged 20 years or older; and individuals who had not used female hormones. The main exclusion criteria included: male participants; individuals under 20 years of age; those who reported using female hormones; and participants with incomplete data. All data analysis and graphic drawing were completed using R4.4.3 software. Restricted cubic spline (RCS) analysis was performed using the RMS package, Bayesian Kernel Machine Regression (BKMR) analysis used the BKMR package, and Shapley Additive Explanations (SHAP) values for Extreme Gradient Boosting (XGBoost) were calculated using the shapforxgboost package.

In this study, we employed a multi-step, multi-method analytical strategy to comprehensively evaluate the associations between sex hormones and FMs. First, logistic regression was applied to assess linear associations in both univariate and multivariate models, while RCS analysis was used to explore potential nonlinear relationships between hormone levels and FM risk. Subsequently, BKMR was employed to evaluate the joint effects of hormones and estimate the Posterior Inclusion Probability (PIP) of individual variables. XGBoost and random forest models were applied to capture complex nonlinear and interactive patterns, with the contribution of each variable interpreted using SHAP analysis. To further assess the robustness of the findings, we performed trend analysis, Least Absolute Shrinkage and Selection Operator (LASSO) regression for variable selection, collinearity diagnostics, and sensitivity analyses. The correlations among hormones and their associations with FMs were visualized using chord diagrams. The integration of multiple analytical approaches not only enhances the robustness of the results but also simultaneously characterizes linear, nonlinear, interactive, and joint effects, thereby providing comprehensive statistical and machine learning support for this study.

### 2.2. Variable definition

Serum levels of E2, E1, FSH, and LH were obtained from the TST questionnaire laboratory data. E2 levels were extracted from the variable LBDESTSI (unit: pmol/L), and E1 levels from LBDESOSI (unit: pmol/L). FSH concentrations were derived from LBXFSH and LH from LBXLUH, both measured in mIU/mL.

FMs status was determined using data from the MCQ Questionnaire. Participants who responded “Breast,” “Cervix (cervical),” “Ovary (ovarian),” or “Uterus (uterine)” in any of the following variables: MCQ230a (first cancer type), MCQ230b (second cancer type), or MCQ230c (third cancer type), were classified as having FMs. Participants who answered “Yes” to MCQ220 (“Ever told you had cancer or malignancy”) but did not report FMs-related cancer types were categorized as having non-FMs cancers. All other responses were excluded from the analysis.

### 2.3. Statistical analysis

#### 2.3.1. Weight adjustment

**Figure 2.**
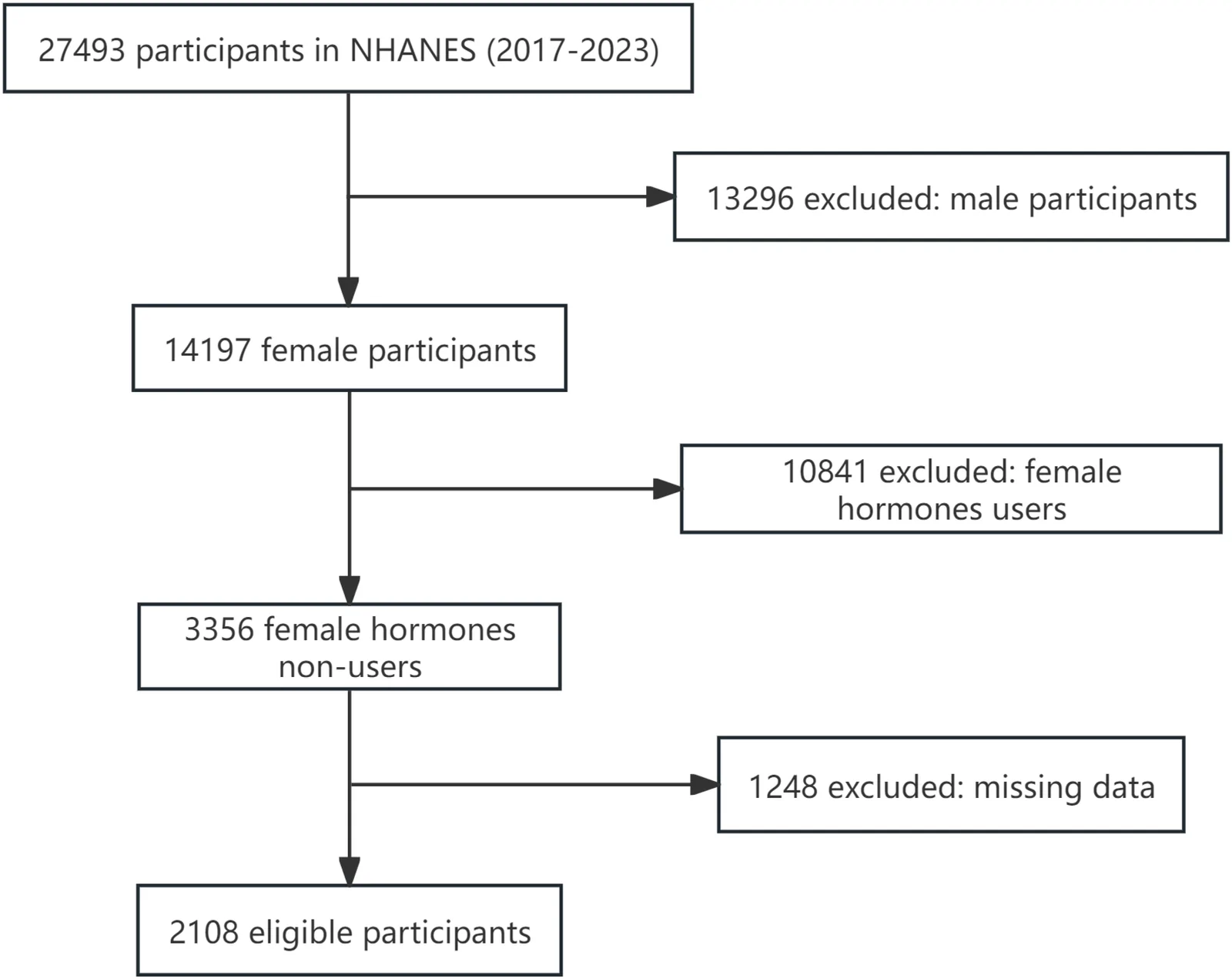
Flowchart of the study design and participant selection. NHANES = National Health and Nutrition Examination Survey.

Based on the inclusion and exclusion criteria, a total of 2108 female participants aged 20 years or older were included in the final analysis after excluding male participants and individuals who reported using female hormones (Fig. 2).

Due to the COVID-19 pandemic, NHANES suspended field operations in March 2020. As a result, data collected from March 2017 until the onset of the pandemic were combined into the 2017 to 2023 survey cycle, and the calculation of sampling weights differed slightly from previous cycles. We followed the official weight adjustment guidelines provided on the NHANES website and applied the recommended procedures to ensure the representativeness and accuracy of the weighted estimates. The method for combining weights across cycles is described as follows:


$$Weight2017\text{-}2020=WTMECPRP\times \frac{3.2}{5.2}$$

$$\begin{array}{c} Weight2021\text{-}2023=WTMEC2YR\times \frac{2}{5.2} \end{array}$$

#### 2.3.2. Baseline analysis and sensitivity analysis

We conducted baseline analyses based on survey design and weighted survey procedures. The Kolmogorov–Smirnov test was used to assess the normality of continuous variables. Variables following a normal distribution were described using mean ± standard deviation (SD) (Mean ± SD), whereas those with non-normal distributions were reported as medians and interquartile ranges (P25, P75). For group comparisons, continuous variables were analyzed using either *t* tests or nonparametric tests, and categorical variables were assessed using chi-squared tests. When expected cell counts were <5, the Rao–Scott adjusted chi-squared test was applied. A *P*-value < .05 was considered statistically significant (Table 1).

**Table 1:** Baseline characteristics of the study population.

Characteristic	N*	Female malignancies			*P* Value‡
		Overall, 2108/38,986,045†	No, 2011/36,385,262†	Yes, 97/2600,783†	
Age	2108	45.00 (32.00, 59.00)	44.00 (32.00, 59.00)	61.00 (51.00, 70.00)	<.001*
PIR	2108	2.07 (1.08, 4.02)	2.06 (1.07, 4.00)	2.27 (1.11, 4.48)	.031*
BMI	2108	29.20 (24.30, 35.20)	29.20 (24.30, 35.30)	28.80 (24.70, 34.60)	.5
WAIST	2108	97.50 (85.80, 111.00)	97.50 (85.50, 111.00)	96.60 (90.30, 110.00)	.2
Estradiol	2108	99.3 (24.30, 346.00)	109 (26.50, 360.00)	24.4 (9.21, 66.80)	.001*
Estrone	2108	166 (96.20, 287.00)	168 (98.20, 292.00)	116 (62.20, 198.00)	.029*
FSH	2108	10 (5.23, 54.70)	9.12 (5.14, 53.40)	50.10 (29.8, 70.30)	<.001*
LH	2108	14 (6.04, 31.00)	13 (5.90, 30.50)	27.20 (17.90, 35.80)	<.001*
Age Category	2108				<.001*
20–40 yr		824 (43%)	813 (45%)	11 (8.3%)	
40–60 yr		761 (35%)	730 (35%)	31 (31%)	
60–80 yr		462 (20%)	413 (17%)	49 (58%)	
80+ yr		61 (2.3%)	55 (2.3%)	6 (3.1%)	
Race	2108				<.001*
Non-Hispanic White		714 (63%)	663 (62%)	51 (84%)	
Non-Hispanic Black		549 (11%)	530 (12%)	19 (5.1%)	
Mexican American		262 (8.4%)	251 (8.8%)	11 (3.3%)	
Other Race		583 (17%)	567 (18%)	16 (7.4%)	
Education	2108				.2
Highschool or less		815 (36%)	772 (35%)	43 (43%)	
College or more		1293 (64%)	1239 (65%)	54 (57%)	
PIR Category	2108				.11
Below poverty line		466 (15%)	447 (16%)	19 (7.5%)	
Low income		553 (20%)	529 (20%)	24 (16%)	
Middle income		558 (30%)	531 (30%)	27 (30%)	
High income		531 (35%)	504 (34%)	27 (47%)	
BMI Category	2108				.0012*
Normal weight		557 (29%)	531 (29%)	26 (26%)	
Underweight		29 (2.0%)	29 (2.2%)	0 (0%)	
Overweight		536 (25%)	510 (25%)	26 (32%)	
Obese		986 (43%)	941 (44%)	45 (42%)	
Waist Category	2108				.0753
Normal		324 (17%)	320 (18%)	4 (6.5%)	
Abdominal obesity		1784 (83%)	1691 (82%)	93 (94%)	
Hypertension	2108	797 (32%)	746 (31%)	51 (51%)	.003*
Diabetes	2108	338 (12%)	319 (11%)	19 (21%)	.047*
Coronary Heart Disease	2108	29 (1.7%)	27 (1.3%)	2 (7.7%)	.3402
Smoking	2108	692 (36%)	650 (35%)	42 (44%)	.2

BMI = body mass index, FSH = follicle-stimulating hormone, LH = luteinizing hormone, PIR = poverty income ratio.

* N not missing (unweighted).

† Median (IQR) for continuous; n (%) for categorical.

‡ Design-based KruskalWallis test; Pearson’s X^2^; Rao & Scott adjustment.

To assess the robustness of the primary findings, multiple sensitivity analyses were performed. First, hormone variables were converted into categorical variables based on clinical reference ranges. Second, Firth logistic regression was used to correct for potential bias. Third, multiple imputation was applied to handle missing data. To minimize the influence of extreme values, the 4 hormone variables were Winsorized by truncating values below the 1st percentile and above the 99th percentile to the corresponding thresholds. Comparisons showed that the mean changes in hormone levels before and after Winsorization were <5%, and the SDs did not differ significantly. The weighted logistic regression results based on the refitted Winsorized data were consistent with those of the main analysis, with no substantial changes in direction or statistical significance, indicating good robustness of the findings. (Table 2).

**Table 2:** Sensitivity analysis of hormone levels before and after Winsorization.

Type	Mean Estradiol	Sd Estradiol	Mean Estrone	Sd Estrone	Mean FSH	Sd FSH	Mean LH	Sd LH
Original data	448	2460	309	1082	31.6	34.1	20.1	17.7
Winsorized data	268	415	233	236	31.5	33.6	19.9	16.8

FSH = follicle-stimulating hormone, LH = luteinizing hormone.

#### 2.3.3. Trend analysis

**Figure 3.**
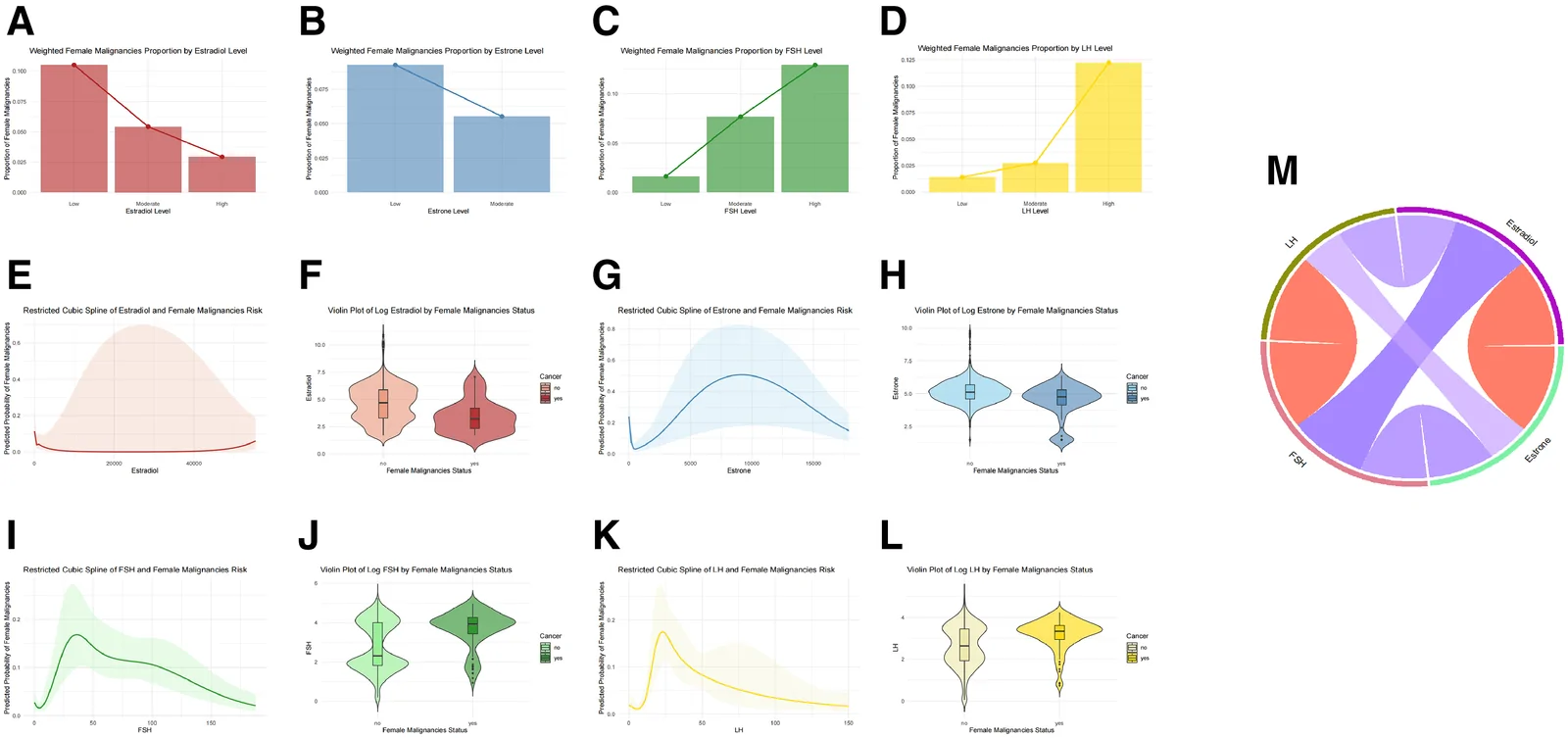
Weighted proportions of female malignancies by hormone levels and nonlinear association and distribution of hormones. All estimates are survey-weighted according to NHANES analytic guidelines. Restricted cubic spline models were adjusted for age, race, body mass index and smoking status. (A) Weighted proportion of female malignancies by Estradiol level. (B) Weighted proportion of female malignancies by Estrone level. (C) Weighted proportion of female malignancies by FSH level. (D) Weighted proportion of female malignancies by LH level. (E) Restricted cubic spline showing the association between estradiol and the risk of female malignancies. (F) Violin plot of log-transformed estradiol by malignancy status. (G) Restricted cubic spline showing the association between estrone and the risk of female malignancies. (H) Violin plot of log-transformed estrone by malignancy status. (I) Restricted cubic spline showing the association between FSH and the risk of female malignancies. (J) Violin plot of log-transformed FSH by malignancy status. (K) Restricted cubic spline showing the association between LH and the risk of female malignancies. (L) Violin plot of log-transformed LH by malignancy status. (M) Chord diagram of estradiol, estrone, FSH, LH, and female malignancy. FSH = follicle-stimulating hormone, LH = luteinizing hormone, NHANES = National Health and Nutrition Examination Survey.

To explore the potential dose–response relationships between SSHs and FMs, continuous hormone variables, including E2, E1, FSH, and LH, were categorized into ordered groups based on clinical reference ranges for trend analysis. Specifically: E2 was divided into 3 groups: low (<73 pmol/L), moderate (73–183 pmol/L), and high (>183 pmol/L); E1 was categorized into 2 groups: low (<110 pmol/L) and moderate (≥110 pmol/L); FSH was classified as low (<10 mIU/mL), moderate (10–25 mIU/mL), and high (>25 mIU/mL); LH was grouped as low (<7 mIU/mL), moderate (7–15 mIU/mL), and high (>15 mIU/mL). These categorized hormone variables were entered into the models as ordinal factors. All models accounted for the complex sampling design of NHANES, incorporating appropriate sample weights, stratification, and clustering variables. Design-adjusted chi-squared tests were performed to further evaluate the statistical associations between hormone categories and cancer outcomes (Table 3, Figures 3A–3D).

**Table 3:** Variance inflation factors (VIFs) of the predictor variables.

Variables	Tolerance	VIF
Estradiol	0.148	6.78
Estrone	0.147	6.80
FSH	0.275	3.64
LH	0.276	3.63

A VIF >10 is generally considered indicative of serious multicollinearity.

FSH = follicle-stimulating hormone, LH = luteinizing hormone, VIF = variance inflation factors.

#### 2.3.4. RCS and data distribution

To further investigate the potential nonlinear associations between SSHs and FMs, RCS regression models were employed under a weighted, complex survey design framework. Following empirical recommendations, 5 percentiles (5th, 27.5th, 50th, 72.5th, and 95th) of each hormone variable: E2, E1, FSH, and LH, were selected as spline knots to construct continuous nonlinear functions. Spline basis functions were then generated for each variable and incorporated into weighted logistic regression models, with FM status as the dependent variable.

The predicted probabilities and their 95% confidence intervals were obtained by applying an inverse logit transformation to the model-derived logit estimates. RCS curves with shaded 95% confidence bands were then plotted to visualize potential nonlinear exposure–response relationships between hormone levels and the risk of FMs. Additionally, to better illustrate the distributional characteristics of each hormone variable, a log-transformation (log + 1) was applied, and hormone distributions stratified by FMs status were visualized using boxplots, violin plots, and swarm plots (Figs. 3E–3l).

#### 2.3.5. Chord diagram

To visualize the relationships among SSHs and their associations with FMs status, we constructed a correlation matrix and generated a chord diagram. Spearman rank correlation coefficients were used to assess pairwise correlations among the 4 hormone variables, while point-biserial correlation coefficients were applied to evaluate associations between each hormone and FMs status. A 5 × 5 correlation matrix was constructed, comprising the 4 SSHs and 1 binary cancer variable. The chord diagram was then created to display the correlation structure. Color mapping in the diagram reflected the strength and direction of the correlations, ranging from –1 to 1: negative correlations were shown in blue, positive correlations in red, and near-zero (neutral) correlations in white. This visualization provided an intuitive overview of both the magnitude and polarity of relationships between variables (Fig. 3M).

#### 2.3.6. Collinearity analysis

To assess the presence of multicollinearity among SSHs, including E2, E1, FSH, and LH, we conducted a collinearity analysis. A linear regression model was first constructed, and multicollinearity was evaluated using 2 standard approaches (Table 3, Table 4):

**Table 4:** Condition indices and eigenvalues for multicollinearity diagnostics.

Dimension	Eigen value	Condition index	Estradiol	Estrone	FSH	LH
2.6860	1.00	0.04158	0.00279	0.003858	0.01557	0.01376
1.8391	1.21	0.00175	0.03447	0.030510	0.00589	0.00408
0.3320	2.84	0.81933	0.00993	0.000305	0.09802	0.02550
0.0731	6.06	0.12159	0.62992	0.636511	0.25678	0.30587
0.0697	6.21	0.01575	0.32289	0.328816	0.62374	0.65078

A condition index above 30 may suggest the presence of severe multicollinearity.

FSH = follicle-stimulating hormone, LH = luteinizing hormone.

Variance inflation factors (VIF): This metric quantifies the degree of linear correlation between each independent variable and the others. A VIF > 10 is generally considered indicative of severe multicollinearity;

Condition Index and Eigenvalues: These metrics assess the collinearity structure of the entire covariance matrix. A condition index >30 typically suggests serious multicollinearity.

#### 2.3.7. Machine learning models

To address the limitations of traditional statistical methods in modeling nonlinear relationships and high-dimensional interactions, we incorporated multiple machine learning approaches, including BKMR, XGBoost, SHAP, LASSO regression, and Random Forest (Supplemental Table 1, Supplemental Digital Content 1).

**Figure 4.**
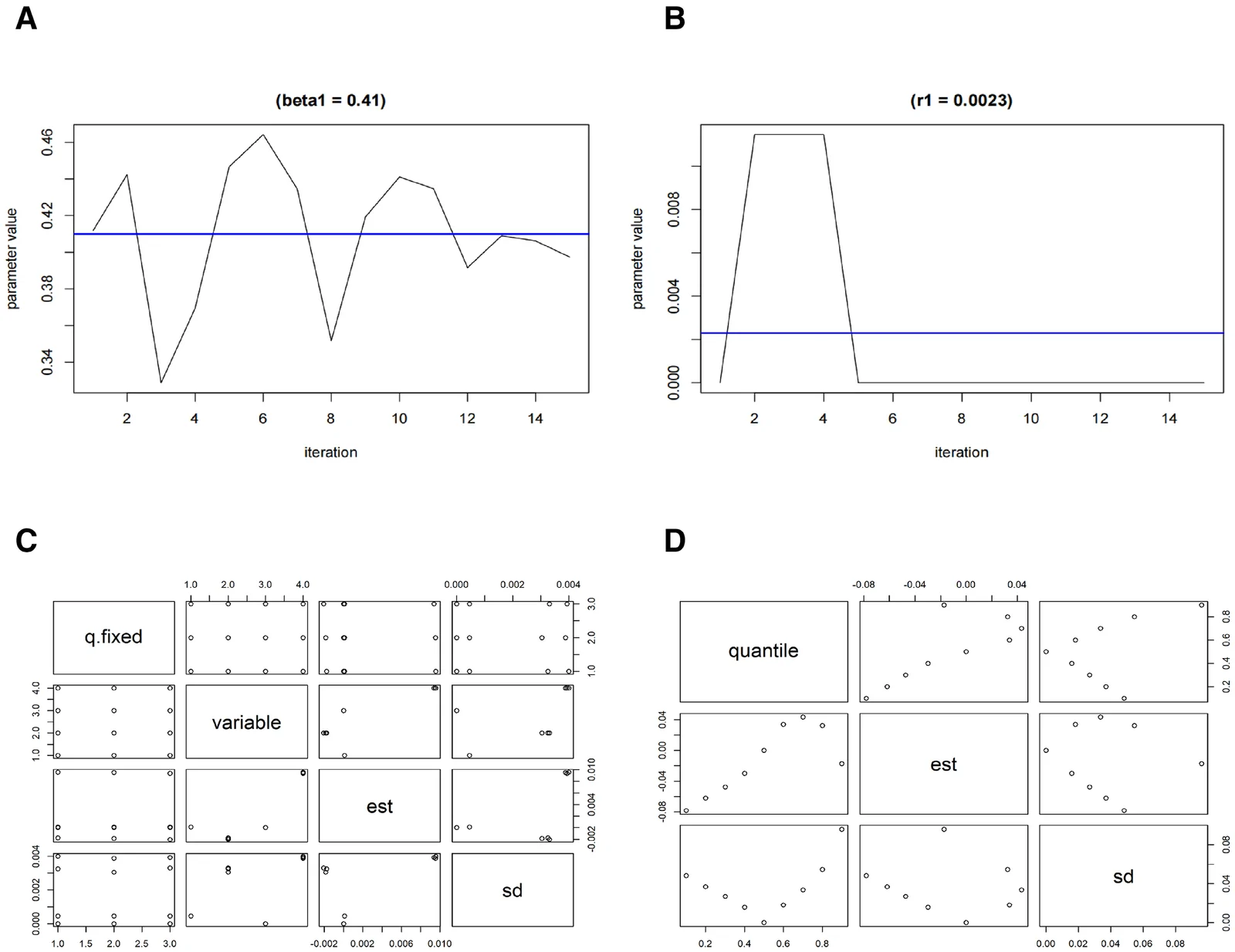
Bayesian Kernel Machine Regression (BKMR) model outputs. The BKMR model was fitted using survey-weighted data and adjusted for the same covariates as the primary multivariable regression models (age, race, body mass index and smoking status). (A) Trace plot of β coefficients. (B) Trace plot of the *r* parameter. (C) Univariate exposure–response curves for each hormone. (D) Overall exposure-response function for the combined hormone mixture.

**Figure 5.**
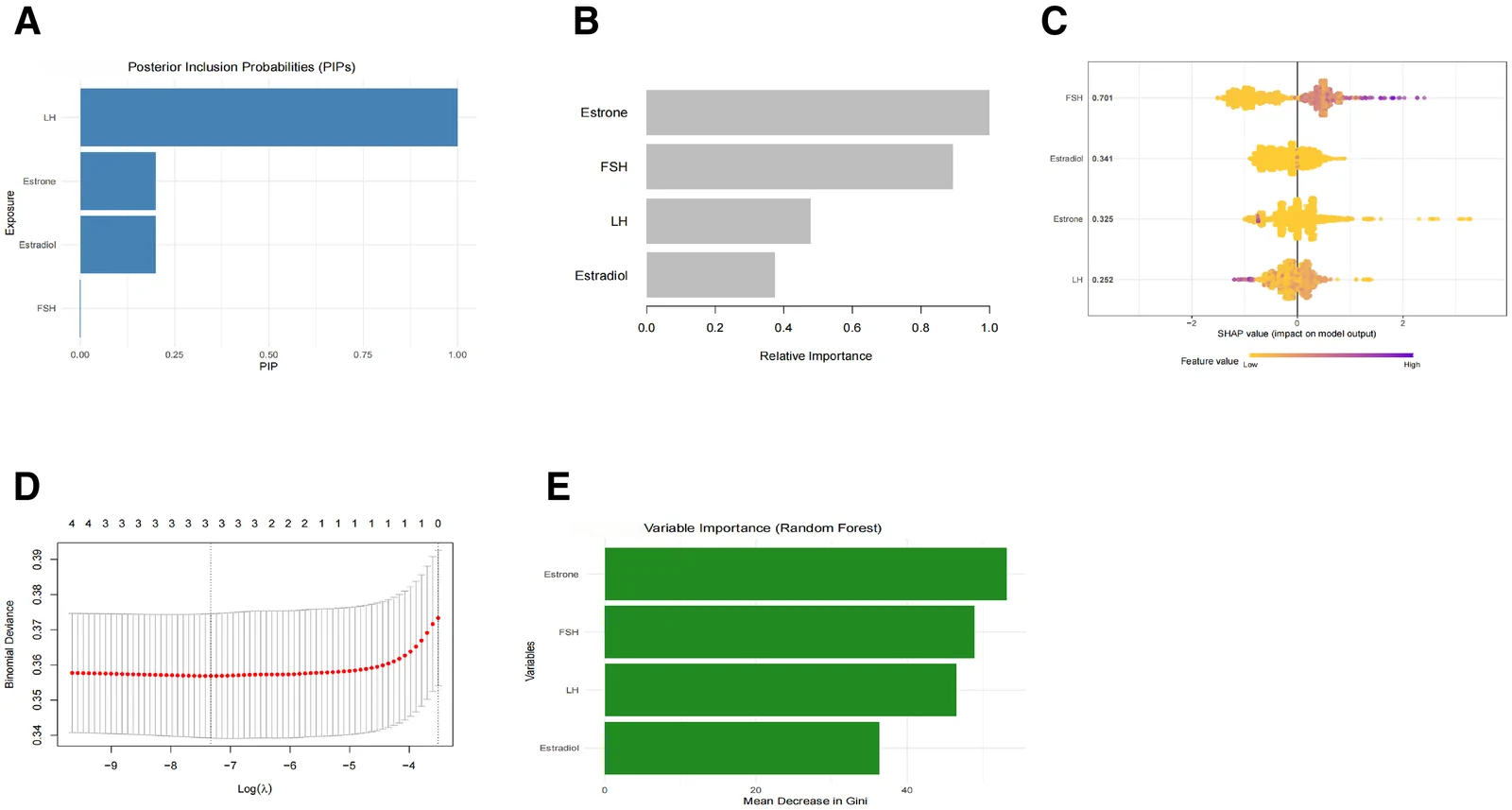
Variable importance and model-derived relevance of sex steroid hormones across multiple analytical frameworks. All machine learning models were trained on survey-weighted data and incorporated the same set of covariates as adjustment variables. Feature importance reflects model-specific metrics and should be interpreted comparatively rather than as causal estimates. (A) Posterior inclusion probabilities (PIPs) of hormone exposures. (B) Feature importance ranking based on the XGBoost model. (C) SHAP summary plot for hormone variables. (D) Cross-validation curve from LASSO logistic regression. (E) Variable importance plot based on the random forest model. LASSO = Least Absolute Shrinkage and Selection Operator, SHAP = Shapley Additive Explanations.

To simultaneously investigate potential nonlinear and interactive effects of multiple SSHs on FMs, we employed BKMR.^[13]^ This method offers several advantages, including the ability to model nonlinear exposure–response relationships, assess the relative importance of high-dimensional exposures, and control for covariate confounding. The BKMR model was specified using a binomial family, and all variables were standardized prior to inclusion. The following outputs were assessed (Figure 4, Figure 5A): Trace plots: Used to monitor the convergence of key parameter estimates (e.g., β coefficients, correlation parameter *r*, and residual variance) during the Markov chain Monte Carlo iterations; Univariate exposure–response curves: Depict the nonlinear associations between individual hormones and cancer risk while adjusting for the other hormones in the model; Overall effect curve: Illustrates how changes in the joint mixture levels of all 4 hormones influence FMs risk; PIP plots: Present the posterior probability of each hormone being included in the model, serving as an indicator of variable importance.

To evaluate the relative contributions of multiple SSHs in predicting FMs, we constructed an XGBoost model. A feature matrix was created and converted into the XGBoost-required DMatrix format. The number of boosting iterations was set to 100. After model training, the relative importance of each variable was extracted and visualized (Fig. 5B). To further interpret the influence of individual hormones on model predictions, we applied the SHAP method to the XGBoost output. Based on game theory, SHAP quantifies the marginal contribution of each variable to the prediction. SHAP values were computed for each variable and sample, and a summary plot was generated to visualize the overall importance ranking and the direction (positive or negative) of each variable’s contribution to the predicted outcomes (Fig. 5C).

To identify the hormone variables with the highest predictive value for FMs, we performed variable selection using LASSO regression. LASSO enables simultaneous variable selection and regularization in high-dimensional data and is effective in addressing multicollinearity. The model was fitted under a logistic regression framework, incorporating both predictors and the binary outcome variable. Ten-fold cross-validation was used to determine the optimal regularization parameter (λ), and a cross-validation error curve was plotted to visualize the selection process. The regression coefficients of variables retained at the minimum λ value (λ_min) were extracted (Fig. 5D).

To further evaluate the nonlinear predictive capability of each hormone variable, we constructed a classification model using the Random Forest algorithm. As an ensemble method that aggregates multiple decision trees, Random Forest offers strong generalization performance and effective variable importance assessment. E2, E1, FSH, and LH were included as predictor variables, and tumor status was used as the outcome variable. The number of trees was set to 500. The model output included the mean decrease in Gini impurity for each variable, which was visualized using a variable importance bar plot (Fig. 5E).

### 2.4. Analytical strategy

To avoid functional overlap among different analytical approaches, the overall analysis strategy was structured in a hierarchical manner. Weighted multivariable logistic regression and RCS models were designated as the primary analytical methods to evaluate the overall associations and potential dose–response relationships between circulating E2, E1, FSH, and LH levels and the risk of malignant tumors in women. Chi-square tests for trend and sensitivity analyses were conducted as supportive analyses to assess the robustness of the primary findings.

BKMR, XGBoost, random forest, LASSO regression, and SHAP were categorized as exploratory analytical tools to characterize nonlinear patterns under combined hormonal exposure, evaluate the relative importance of individual hormones, and explore heterogeneity in individual-level predictions. Although BKMR is capable of assessing interaction effects, it was primarily used in this study to describe the overall joint exposure effects and to quantify variable contributions using posterior inclusion probabilities PIP; interaction results were interpreted descriptively rather than for formal hypothesis testing. Overall, the machine learning–based methods were intended to complement conventional regression analyses by providing hypothesis-generating insights rather than supporting causal inference.

## 3. Results

### 3.1. Weighted analysis

Baseline characteristics were analyzed for the total study population of 2108 participants, which represented 38,986,045 individuals in the weighted U.S. population. Among them, 97 individuals were identified as having FMs, corresponding to an estimated 2600,783 individuals nationwide, while 2011 individuals without FMs represented 36,385,262 individuals. The median age in the FMs group was 61.00 years (range: 51.00–70.00), compared to 44.00 years (32.00–59.00) in the non-FMs group. Non-Hispanic White participants comprised the majority in both groups (FMs: 51 [84.00%]; non-FMs: 663 [62.00%]). Significant differences in hormone levels were observed between the FM and non-FM groups. Compared with non-FM participants, those with FMs exhibited lower levels of E2 and E1, but higher levels of FSH and LH (all *P* < .05). These associations remained significant after applying survey weights, suggesting that decreased estrogen levels and elevated gonadotropins may be linked to an increased risk of FMs (Table 1).

To evaluate the influence of extreme values on hormone measurements, we conducted a Winsorization-based sensitivity analysis (Table 2). The results showed that the means and SDs of E2 and E1 decreased markedly after the removal of extreme values. Specifically, the mean E2 level decreased from 448 to 268, and the SD decreased from 2460 to 415, indicating the presence of pronounced outliers in the raw data that affected the central tendency. In contrast, changes in the mean and SD for FSH and LH were minimal, suggesting that their distributions were more stable and less affected by extreme values. Overall, the core hormone variables remained consistent after Winsorization, supporting the robustness of the study findings.

To examine the potential dose–response relationships between SSHs and FMs, a trend analysis was conducted. As illustrated in Figures 3A–3D, the weighted trend analysis revealed distinct directional patterns between hormone levels and FMs prevalence. E2 levels showed a negative trend, with higher concentrations associated with a lower incidence of FMs. E1 exhibited a similar downward trend; however, due to the absence of a high-level group, the decline was only observed from low to moderate levels. In contrast, FSH and LH demonstrated strong positive trends, with FM risk increasing alongside hormone concentrations. These findings suggest that gonadotropins may play a promotive role in the development of FMs, whereas estrogens may exert a potential protective effect. It should be noted that these group-level comparisons and trend analyses reflect average distributional differences across exposure categories and do not preclude the presence of nonlinear or threshold effects when hormone levels are modeled as continuous variables.

To further examine the potential nonlinear relationships between SSHs and the risk of FMs, RCS models were applied. The RCS curve for E2, with shaded 95% confidence bands, indicated a nonlinear trend in the association with FM risk, showing elevated predicted probabilities at both lower and higher hormone concentrations compared with moderate levels. Moreover, patients with FMs exhibited significantly lower serum E2 levels than those without FMs, indicating that reduced E2 concentrations may be associated with increased risk. For E1, the RCS analysis demonstrated a nonlinear association with FM risk, in which the predicted probability was higher at moderate concentrations and lower at both extremes, as shown within the 95% confidence bands. Although E1 levels were slightly lower in the FMs group than in the non-FMs group, the difference was minimal, suggesting a limited contribution of E1 to FMs risk. In contrast, FSH exhibited a pronounced nonlinear association with FMs risk. The predicted probability increased sharply at lower concentrations and gradually decreased thereafter. Moreover, patients with FMs had substantially higher FSH levels than non-FMs participants, implying that elevated FSH concentrations might be linked to an increased risk of FMs. A nonlinear association was also identified between LH levels and the risk of FMs. The predicted probability of FMs peaked at approximately 30 to 40 mIU/mL and gradually declined thereafter. This pattern suggests that LH may exert concentration-dependent effects on FMs risk, with moderate levels associated with the highest predicted probability (Figs. 3E–3L). Collectively, these findings suggest that SSHs may exert complex and dose-dependent effects on FMs risk, warranting further mechanistic investigation.

To further explore the interrelationships among the 4 hormone variables, a chord diagram was constructed (Fig. 3M). The results indicated that E2 and E1 exhibited the strongest positive correlation, as evidenced by the widest connecting chord. A notable positive association was also observed between FSH and LH. In addition, moderate cross-links were observed between the estrogenic hormones (E2 and E1) and the gonadotropins (FSH and LH).

To assess multicollinearity among the independent variables in the model, we performed collinearity diagnostics using 2 approaches: VIF and the condition index (Tables 3 and 4). All 4 hormone variables had VIF values below the commonly accepted threshold of 10: E2 (VIF = 6.78), E1 (VIF = 6.80), FSH (VIF = 3.64), and LH (VIF = 3.63), indicating no evidence of severe multicollinearity. However, the VIF values for E2 and E1 were close to 7, suggesting a moderate degree of collinearity between them. In contrast, the lower VIF values of FSH and LH (<4) implied minimal multicollinearity risk. The results of the Condition Index analysis further supported these findings. None of the condition indices exceeded the critical threshold of 30, indicating an overall absence of severe multicollinearity. However, in the fourth dimension, a simultaneous elevation in variance decomposition proportions was observed for E2, E1, FSH, and LH, with E2 and E1 showing particularly high proportions (0.63 and 0.64, respectively). This pattern suggests a potential collinearity structure between E2 and E1 in the model. These findings are consistent with the chord diagram (Fig. 3M).

### 3.2. Machine learning models

To assess the joint and nonlinear effects of SSHs exposure on the risk of FMs, we applied BKMR analysis (Fig. 4). The trace plot of the β parameters indicated that the posterior estimates stabilized after approximately 15 iterations, demonstrating adequate model convergence and fit (Fig. 4A). The trace plot of the r parameter remained close to zero, suggesting limited interaction effects among hormones and a model structure primarily driven by main effects (Fig. 4B). Univariate exposure–response analyses revealed that for both estrogenic and gonadotropic hormones, certain variables: such as E2 and LH: were associated with increased FMs risk at higher concentrations (Fig. 4C). The overall effect plot further supported a positive dose–response pattern, showing that FMs risk progressively increased with higher cumulative hormone exposure (Fig. 4D). PIP analysis identified LH as the most predictive variable in the model (PIP = 1.0), with substantially greater importance than E2 and E1, while FSH contributed minimally (Fig. 5A). These results suggest that LH may play a central role in the development of FMs.

To further investigate the predictive importance of each hormone variable for FMs, we developed an XGBoost model and visualized the relative importance of each feature (Fig. 5B). The results indicated that among the 4 included hormones, E1 had the highest relative importance, followed by FSH, LH, and E2, suggesting that E1 contributed most to FMs risk prediction within this nonlinear tree-based model. To enhance model interpretability, we further applied SHAP to decompose prediction values at the individual-level (Fig. 5C). The SHAP analysis revealed that FSH had the highest mean SHAP value (0.701), indicating the strongest independent impact on model output (i.e., FMs prediction). E2 and E1 followed with mean SHAP values of 0.341 and 0.325, respectively, while LH had the lowest contribution (0.252), suggesting a relatively weaker overall influence. Additionally, the SHAP summary plots demonstrated heterogeneity in the direction of variable effects. Higher FSH values were frequently associated with positive SHAP values, implying that increased FSH levels may elevate FM risk. In contrast, the SHAP distributions for E2 and E1 were more dispersed, with both high and low values contributing to either positive or negative impacts on the prediction. This pattern suggests that their effects on FMs risk may be nonlinear or subject to individual-level variation.

To further identify key hormone variables associated with FMs, we performed feature selection using the LASSO regression and visualized the cross-validation error curve (Fig. 5D). The results showed that when log(λ) ≈ –7, the binomial deviance reached its minimum, and 3 variables were retained in the model, indicating optimal predictive performance and minimal risk of overfitting at this penalty level. In parallel, we applied a Random Forest model to assess the importance of hormone variables (Fig. 5E). The results indicated that E1 contributed the most to classification performance, followed by FSH and LH, while E2 demonstrated the lowest relative importance. This finding was consistent with the results of the XGBoost model, further confirming that E1 exhibits strong and stable predictive power across multiple nonlinear algorithms. Differences in key hormones identified across machine learning models likely reflect differences in model structure, optimization objectives, and variable importance metrics, and should therefore be interpreted as exploratory, hypothesis-generating findings rather than confirmatory evidence.

## 4. Discussion

In this study, using nationally representative NHANES cross-sectional data, we systematically examined the associations between SSHs (E2, E1, FSH, and LH) and FMs. The results showed that higher levels of LH and FSH were positively associated with FMs, whereas estrogenic hormones (E2 and E1) exhibited generally negative or nonlinear associations. Trend analyses and RCS modeling revealed dose–response relationships for gonadotropins and complex nonlinear patterns for estrogens. Multiple analytical approaches, including weighted logistic regression, BKMR, XGBoost, Random Forest, SHAP, and LASSO, consistently identified E1, FSH, and LH as key predictors: LH had the highest PIP in BKMR (PIP = 1.0), FSH showed the highest mean SHAP value, and E1 had the greatest relative importance in both XGBoost and Random Forest. Moderate correlations among hormones indicated the need to account for multicollinearity during modeling. Overall, LH, FSH, and E1 demonstrated high predictive consistency and explanatory power across diverse analytical frameworks, highlighting their potential value in associations stratification for FMs.

The high E2 levels observed in this study may exert a potential protective effect, which differs from the findings reported in previous cohort and meta-analytic studies on hormone-related cancers. For example, a case-cohort analysis reported that higher circulating sex steroid hormone levels were associated with an increased risk of postmenopausal estrogen receptor–positive breast cancer (BC).^[14]^ Similarly, a recent systematic review and meta-analysis suggested that endogenous sex hormones may contribute to the development of hormone-related malignancies such as endometrial cancer.^[15]^ This discrepancy may be largely attributed to the study by Zhang et al focusing on postmenopausal women, whereas our study targeted women of full reproductive age.^[16]^ This difference may reflect distinct physiological mechanisms of hormonal regulation between premenopausal and postmenopausal women, as substantial changes occur in estrogen levels and in the feedback regulation of the hypothalamic–pituitary–gonadal axis across different reproductive stages. In addition, nonlinear models (RCS and BKMR) were employed in this study to identify threshold and saturation effects that are difficult to capture using conventional linear models. Therefore, our findings extend previous evidence by suggesting that the association between estrogen and female cancer is not fixed but may depend on an individual’s hormonal milieu and physiological stage. Nevertheless, potential confounding factors such as postmenopausal status, age, and obesity may influence the observed associations between hormone levels and FMS.^[17–20]^ Therefore, the results of this study should be interpreted with caution.

As this study represents a secondary analysis of an existing population-based dataset, the sample size was determined by data availability rather than a priori power calculations. Accordingly, interpretation of the findings emphasizes effect estimates, confidence intervals, and the consistency of results across multiple sensitivity analyses. Importantly, the results should be understood within a descriptive and associative framework. Given the cross-sectional design and the use of self-reported, pooled female malignancy outcomes, this study does not aim to establish causal relationships, risk prediction models, or validated biomarkers. Instead, its primary contribution lies in characterizing complex, model-dependent patterns of association between circulating sex steroid hormone levels and a history of FMs. Differences in “key hormones” identified across traditional regression models and machine learning approaches are explicitly acknowledged and interpreted as reflecting the sensitivity of distinct analytical frameworks to different data features, rather than as mutually validating or convergent evidence of biological primacy.

E2 is the most biologically active form of endogenous estrogen. It exerts its effects primarily by activating estrogen receptors, including ERα and ERβ.^[21]^ In addition, E2 can promote angiogenesis in the breast tumor microenvironment by upregulating VEGF and facilitate immune evasion by suppressing T cell activity. It is also involved in the accumulation of deoxyribonucleic acid damage and inhibition of deoxyribonucleic acid repair mechanisms.^[22,23]^ Environmental exposures may also influence hormone-related cancer risk through endocrine disruption. For example, analyses of NHANES data have demonstrated associations between heavy metal exposure and pan-cancer risks related to sex hormone dysregulation.^[24]^ In this study, E2 exhibited a complex nonlinear pattern in both RCS and BKMR analyses, suggesting that its effect may be influenced by hormone concentration, receptor expression levels, menopausal status, and interactions within the tumor microenvironment.

E1 is the predominant form of circulating estrogen in postmenopausal women, primarily derived from the peripheral conversion of androstenedione by the aromatase enzyme (CYP19A1) in adipose tissue.^[25–27]^ E1 can be converted back into E2 by 17β-HSD1, thereby sustaining local estrogen signaling, a process that is particularly pronounced in ER-positive tumors.^[28–30]^ In addition, epidemiological analyses using NHANES data have suggested that reproductive factors such as age at menopause may reflect cumulative estrogen exposure and are associated with the risk of gynecologic cancers, further highlighting the importance of long-term hormonal milieu in FMs.^[31]^

FSH is secreted by the anterior pituitary and traditionally functions to stimulate follicular development and hormone synthesis. Studies have shown that elevated FSH levels are associated with an increased risk of BC, particularly during the early postmenopausal period, when fluctuations in FSH levels closely coincide with the dynamics of BC incidence.^[9,32]^ Although LH primarily regulates ovulation and corpus luteum formation, it has also been implicated in tumorigenesis. LH can activate ovarian steroidogenic pathways and indirectly modulate estrogen levels. In addition, its receptor (LHCGR) has been found to be expressed in certain BC cells, suggesting a potential direct oncogenic role.^[33,34]^ In this study, LH exhibited the highest PIP in the BKMR analysis, indicating a strong, independent, and stable contribution to risk prediction.

This study, based on nationally representative data from NHANES, employed a combination of statistical and machine learning approaches to systematically analyze the association between sex hormones and BC, demonstrating both broad applicability and methodological rigor. Cross-validation across multiple models enhanced the robustness of the findings and revealed differential contributions of various hormones under different modeling frameworks.

However, this study has certain limitations. First, sex hormone levels were measured at a single time point, which may not accurately capture long-term exposure or temporal variability. Although a single measurement is inherently imperfect, previous studies have demonstrated that it can still serve as a reliable predictor of long-term associations with BC. Moreover, the use of large population-based samples can statistically mitigate random measurement error to some extent.^[16]^ Second, this study employed a cross-sectional design, which limits the ability to infer potential mechanisms or causal relationships. The findings should be regarded as exploratory and hypothesis-generating, with mechanistic interpretations explained cautiously and solely to situate the observed associations within plausible biological contexts. In addition, the diagnosis of FMS in NHANES was based on self-reported information, which may have introduced recall and misclassification biases. Since NHANES does not validate self-reported diagnoses using gold-standard clinical methods, there is a risk of both false-positive and false-negative classifications. Future research should adopt prospective cohort designs and randomized controlled trials with clinically confirmed diagnoses to more effectively establish causal relationships and minimize potential sources of bias.

## 5. Conclusion

Based on NHANES data, this study systematically evaluated the associations between 4 SSHs: E2, E1, FSH, and LH, and the risk of FMs. The results suggest that elevated levels of FSH and LH were consistently associated with a higher likelihood of self-reported FMs, while E2 and E1 exhibited more complex nonlinear patterns. Although the ranking of variable importance varied across models, the overall trends were generally consistent. In summary, SSHs showed robust associations across multiple analytical frameworks, without implying direct etiological roles. This cross-sectional analysis highlights notable correlations between gonadotropins and female malignancy history, which should be further investigated in longitudinal studies with clinically verified outcomes.

## Acknowledgments

We are sincerely grateful for the unwavering support and valuable guidance provided by our supervisors, the commitment demonstrated by the study participants, and the enduring encouragement from our families and friends. We also recognize the essential contributions of scholars and researchers whose influential work has significantly informed and enriched the development of this study.

## Author contributions

**Formal analysis:** Ningning Ren.

**Visualization:** Ningning Ren.

**Resources:** Mengjie Yang, Fangyu Zhou.

**Methodology:** Jiamin Ma, Wenzhong Ji.

**Conceptualization:** Xiaoyi Ren.

**Supervision:** Xiaoyi Ren.

**Writing – original draft:** Ningning Ren.

**Writing – review & editing:** Xiaoyi Ren.


