# Supplementary material for: Associations between sex steroid hormones and female malignancies: A cross-sectional analysis of NHANES data
Source: Medicine (Baltimore). 2026 Aug 21;105(34):e50317. doi: 10.1097/MD.0000000000050317 (PMC13502748; doi:10.1097/MD.0000000000050317)
Supplement: Supplementary file 1 [file medi-105-e50317-s001.docx]

**Supplemental Table1: Variable importance metrics for estradiol, estrone, FSH, and LH in predicting Cancer using XGBoost and Random Forest models.**

| **Variable** | **XGBoost**  **Gain** | **XGBoost Cover** | **XGBoost Frequency** | **Random Forest MeanDecreaseGini** |
| --- | --- | --- | --- | --- |
| **Estradiol** | 0.45 | 0.40 | 0.35 | 0.25 |
| **LH** | 0.30 | 0.25 | 0.30 | 0.20 |
| **FSH** | 0.15 | 0.20 | 0.20 | 0.15 |
| **Estrone** | 0.10 | 0.15 | 0.15 | 0.10 |
